# Serum folate receptor α (sFR) in ovarian cancer diagnosis and surveillance

**DOI:** 10.1002/cam4.1944

**Published:** 2019-02-13

**Authors:** Batoul Farran, Samet Albayrak, Judith Abrams, Michael A. Tainsky, Nancy K. Levin, Robert Morris, Larry H. Matherly, Manohar Ratnam, Ira Winer

**Affiliations:** ^1^ Department of Oncology Wayne State University and Karmanos Cancer Institute Detroit Michigan; ^2^ Department of Obstetrics and Gynecology Wayne State University Detroit Michigan; ^3^ Department of Oncology Wayne State University and Karmanos Cancer Institute and Center for Molecular Medicine and Genetics Detroit Michigan; ^4^ Department of Oncology, Division of Gynecologic Oncology Wayne State University and Karmanos Cancer Institute Detroit Michigan; ^5^ Departments of Oncology and Pharmacology Wayne State University and Karmanos Cancer Institute Detroit Michigan

**Keywords:** biomarkers, gynecologic oncology, translational research

## Abstract

Novelty and Impact Statement: Our findings suggest that soluble folate receptor (sFR) could be used in both the initial diagnosis and surveillance of patients with ovarian cancer. Our cohort constitutes one of the largest comparison groups for sFR analyzed so far. We have defined the background level of sFR using healthy volunteers. This is also the first study to prospectively follow patients in the surveillance setting to concurrently identify differential changes in tumor markers CA‐125 and sFR.

## 1 INTRODUCTION

Ovarian cancer (OVCA) constitutes the deadliest gynecologic malignancy, with ~14 000 deaths anticipated in the United States in 2017.^1^ Although epithelial cancers display response rates of 80% to standard therapy, up to 70% of patients relapse within 2 years. This poor prognosis is due to lack of early detection, as well as to innate and acquired resistance to chemotherapy.^2^, ^3^, ^4^, ^5^ Specifically, while early‐stage OVCA is often cured by surgery, <30% of OVCAs are detected in stage 1, with >60% diagnosed at Stage 3/4. Current biomarkers (eg CA125, HE‐4) and detection efforts with ultrasound and physical examination have fallen short of effective early diagnosis. Efforts to identify panels of biomarkers via transcriptomics and proteomics have likewise failed to produce significant advances. Predicting which patients are likely to recur or identifying recurrence early is also a significant challenge with treatment implications. Novel approaches to diagnostics and therapeutics are therefore required. One biomarker which has received significant attention is folate receptor α (FR). Folate receptor α is a glycophosphatidylinositol (GPI) anchored glycopolypeptide.^6^ It is limited to luminal surfaces in normal epithelial cells but is highly expressed in nonmucinous OVCA. In 2014, a study evaluating 2801 patients from the Ovarian Tumor Tissue Analysis (OTTA) consortium linked to data from the Cancer Genome Atlas (TCGA) established that FR was overexpressed in 76% of high‐grade serous ovarian carcinoma (HGSC).^4^ A substantial portion of FR is released into the blood in soluble form (sFR), either via a membrane‐associated protease or a GPI‐specific serum phospholipase,^7^, ^8^ while normal serum contains virtually no detectable sFR. There are limited data linking high sFR to shorter progression‐free survival (PFS).^9^

To assess whether sFR constitutes a clinically relevant biomarker of OVCA suitable for diagnosis and surveillance, we investigated one of the largest cohorts to date utilizing serum FR levels, including healthy controls and patients with benign conditions and OVCA. Additionally, we prospectively followed a group of patients after initial diagnosis in the surveillance setting which, as yet, has not been described in the literature for sFR. Our aims were to: (a) validate the extent to which sFR can distinguish between healthy, benign and OVCA patients; and (b) evaluate the ability of sFR to predict early disease recurrence.

## 2 MATERIALS AND METHODS

### 2.1 Patient serum

Whole blood obtained from 130 healthy controls, 92 patients with benign disease, 14 patients with disease of low malignant potential (LMP), and 180 patients with OVCA of all stages (68% with HGSC) was centrifuged at 2500 rpm at 4°C for 15 min and supernatants were stored at −80°C. sFR was measured in all samples, and CA125 was measured via ELISA (Syntron Bioresearch, Carlsbad, CA) in 44 healthy patients. Samples were obtained from patients at Karmanos Cancer Center, St. John Hospital and Oakwood hospital in Detroit, MI, and at the Mayo Clinic, Rochester, MN. Additional specimens were provided by the Cooperative Human Tissue Network (CHTN) and GOG specimen banks, prior to original surgery or therapy. Healthy patients’ specimens were obtained from outreach sites or clinics in the Detroit area under the current IRB guidance. CA125 levels from 88 patients with OVCA were also measured. Similarly, whole blood was obtained from 28 patients with HGSC OVCA at Karmanos Cancer Center during surveillance and serum was obtained. sFR levels were measured, and CA125 was abstracted from clinical charts. Charts of OVCA patients were abstracted for demographic information, pathology and clinical course. Healthy volunteers were self‐reported to be disease‐free. Protocols were approved by the Institutional Review Boards of Wayne State University and the individual hospitals.

### 2.2 sFR binding studies

Soluble folate receptor α was quantified in serum samples by measuring [^3^H]folic acid binding, as described.^10^ Standard curve calibration was performed. Bovine folate‐binding protein (Sigma Aldrich, St Louis, MO) was used as a sFR surrogate. Normal sera from healthy volunteers were utilized as controls. Serum samples (10 µL) were diluted into 0.5 mL of buffer (10 mmol/L sodium phosphate buffer (pH 7.5)/150 mmol/L NaCl/1% Triton X‐100). [^3^H]Folic acid (Moravek, Brea, CA) (2 pmol) was added and incubated for 1 hour at 37°C. Protein‐bound [^3^H]folate was measured by charcoal binding.^10^ Nonspecific [^3^H]folate binding was determined in parallel with diluted serum treated for 10 minutes with 100× excess of unlabeled folic acid (200 pmol).

### 2.3 Statistical methods

For evaluation of sFR as a biomarker, logistic regression was used to model the probability of OVCA as a function of sFR levels. Discrimination (c statistic) was estimated as the area under the ROC curve (AUC) with bootstrapped 95% confidence intervals, using fivefold cross‐validation. Sensitivity and specificity were estimated using predicted probabilities from logistic models. The discriminant slope was calculated as the difference between the mean probabilities of the outcome for those with and without the outcome.

Associations between CA125 and sFR were assessed with Spearman's rank correlation coefficient. Background values were subtracted from each sFR measurement, and the mean of two values was used in the analyses.

## 3 RESULTS

### 3.1 Standardization of sFR protocol/binding assay

We optimized the sFR binding assay prior to evaluating serum samples from OVCA patients. sFR measurements were performed in quintuplicate using serum samples from three healthy volunteers into which known quantities of bovine folate‐binding protein were added. A volume of 10 µL of serum was sufficient to yield reproducible results and 2 pmol of [^3^H]folic acid was used. The optimized assay was accurate and sensitive to a level of <5 fmol in 10 µL of serum (Figure S1).

### 3.2 sFR identifies women with malignant conditions

The diagnostic utility of sFR was evaluated in a cohort of 416 patients, including patients diagnosed with benign disease (n = 92), low malignant potential OVCA (LMP, n = 14), and OVCA of all stages (n = 180), most of whom had HGSC (68%). Sera from healthy volunteers (n = 130) were utilized to establish a background level for sFR. Patient demographics for OVCA patients were similar, including the healthy volunteers, and are summarized in Table S1. The median age was 59 years old. The majority of patients were self‐identified as Caucasian (81% for ovarian cancer cohorts vs 90% for healthy patients); there were 6% African American patients, with the remainder of Asian American or “other” race. For the benign patients, the average age was slightly younger at 48 years old, and 80% of the cohort were Caucasian patients; there were 10% African American patients, with the remainder Asian American or “other.” Healthy volunteers were self‐reported to be free of disease, and follow‐up interviews were performed randomly for ~40% (50/130) of these patients within one to four years after serum collection to confirm no new malignancies or benign health conditions.

**Figure 1:**
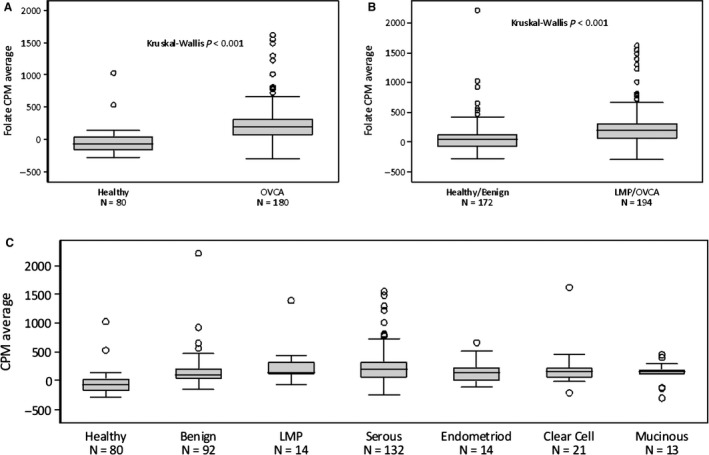
“Initial Diagnosis” Cohort: sFR levels. A, sFR levels for healthy women (n = 80) vs women with ovarian cancer/LMP (n = 194), using the Kruskal‐Wallis test. Healthy women differ from those with OVCA (*P* < 0.001). B, sFR levels for healthy women and those with benign disease (n = 172) compared to women with LMP tumors and ovarian cancer. Healthy/benign vs. OVCA results were significantly different (*P* < 0.001). C, sFR levels were broken down by tumor type for histologic subtype. Healthy patients are different from all subtypes of cancer, as well as benign (*P* < 0.001). Benign results are significantly different from those for HGSC (*P* < 0.0002) but not from other subtypes (given smaller n‐values)

Sixty‐seven percent of healthy women had sFR levels that did not exceed background, compared to 15% of patients with OVCA, LMP, or benign conditions. When considering healthy women versus those with OVCA, we saw a significant difference in the average level of sFR (*P* < 0.001); when combining healthy women and those with benign disease together as controls versus patients with LMP and OVCA, these differences were still significant (Table S2, Figure 1A/B; *P* < 0.001).

When sFR levels were evaluated according to histologic type (Figure 1C), a statistically significant difference between sFR in HGSC, compared to both healthy controls and benign patients, was noted (*P* = 0.002). There were no significant differences noted between the other histologic subtypes (ie mucinous, clear cell, and endometriod) and benign patients and healthy controls; however, this analysis is significantly limited given small numbers in these other cohorts. We further evaluated sFR as a screening biomarker to distinguish healthy women from those with OVCA; sensitivity was high (>90%), but specificity was poor (59%; Tables 1 and S3). The AUC was 0.87 (95% CI: 0.82, 0.92). The false‐negative rate in this setting was <15% except for clear cell (23%), secondary to sample size. When comparing healthy women and those with benign conditions to combined LMP/OVCA without distinguishing subtype (Table 1A), sensitivity and specificity were 71% and 65%, respectively. The AUC was 0.72 (95% CI: 0.67, 0.78), and the false‐negative rate in this comparison was <30% for all tumor types except clear cell (36%). When analyzed by histology (Table 1B), and compared separately to healthy individuals and those with benign conditions, sensitivity for HGSC approached >90% and specificity for the other types was correspondingly high (although sensitivity is low).

**Table 1:** sFR as a screening biomarker. Logistic regression was used to model the probability of ovarian cancer (OVCA) as a function of sFR. Discrimination (c statistic) was estimated as the area under the ROC curve (AUC) with bootstrapped 95% confidence intervals using fivefold cross‐validation. Sensitivity and specificity were estimated using a cut point of 50%. The discriminant slope was calculated as the difference between the mean probabilities of the outcome for those with/without the outcome. In (A), specificity and sensitivity are calculated based on the comparator in the second column not on histologic type, but rather on overall grouping (“OVCA” or “LMP/OVCA”). In (B), sensitivity and specificity are calculated as noted based on OVCA histologic type

(A) Sensitivity/specificity of sFR in healthy controls vs OVCA without distinction by subtype			
	Sensitivity (95% CI)	Specificity (95% CI)	AUC (95% CI)
LMP/OVCA			
Healthy/benign	71% (65%, 78%)	65% (58%, 72%)	0.72 (0.67, 0.78)
Benign	100%	0%	0.60 (0.54, 0.67)
OVCA			
Benign	100%	0%	0.60 (0.53, 0.67)
Healthy	91% (86%, 94%)	59% (48%, 69%)	0.87 (0.82, 0.92)

(B) Sensitivity/specificity of sFR by OVCA histologic type			
	N	Sensitivity (95% CI)	Specificity (95% CI)
Serous OVCA/LMP vs	132		
Healthy/benign	172	45% (36%, 53%)	85% (79%, 90%)
Healthy	80	86% (79%, 91%)	69% (58%, 78%)
Benign	92	94% (88%, 97%)	2% (1%, 8%)
Endometrioid OVCA/LMP) vs	21		
Healthy/benign	172	5% (1%, 23%)	99% (97%, 100%)
Healthy	80	24% (11%, 45%)	98% (91%. 99%)
Benign	92	0% (0%, 15%)	100% (96%, 100%)
Clear Cell OVCA/LMP vs	14		
Healthy/benign	172	0% (0%, 22%)	100% (96%, 100%)
Healthy	80	14% (4%, 40%)	98% (91%, 99%)
Benign	92	0% (0%, 22%)	100% (96%, 100%)
Mucinous OVCA/LMP vs	13		
Healthy/benign	172	0% (0%, 23%)	98% (91%. 99%)
Healthy	80	0% (0%, 23%)	98% (91%, 99%)
Benign	92	0% (0%, 23%)	100% (96%, 100%)

To evaluate the association between sFR and CA125, we correlated the levels of sFR and CA125 at initial diagnosis in a smaller cohort (n = 44 benign/healthy and n = 88 with OVCA). The association was weak, although statistically significant (Spearman's rho = 0.28, *P* = 0.0008; Figure S2). Both retained their ability to distinguish healthy women from women with OVCA when entered simultaneously into a logistic model (sFR: *P* < 0.001, CA125 *P* = 0.005).

Specifically, the odds of having OVCA increased by 53% for each 100 units of CA125 and by 93% for each 100 units of sFR.

### 3.3 sFR in OVCA surveillance

**Figure 2:**
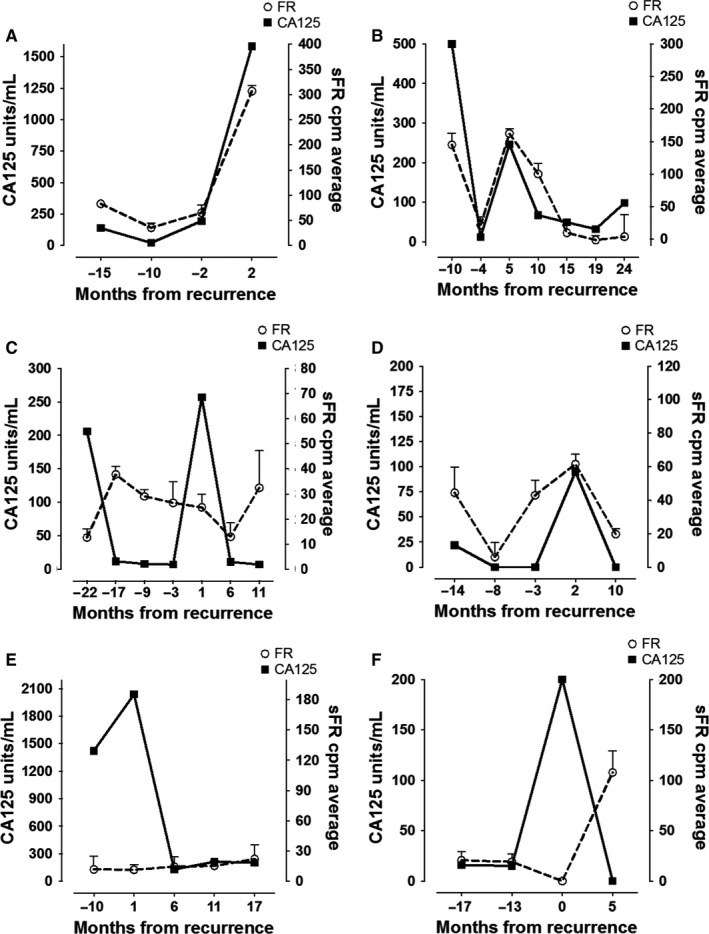
sFR/CA125 in Surveillance cohort. A small, pilot cohort of patients was monitored prospectively for descriptive purposes. Examples of patient sFR and CA125 levels following initial treatment at different time intervals prior to and following recurrence are depicted. The *y*‐axis depicts sFR and CA125 levels (each with its own axis given differing levels); the *x*‐axis depicts time. A “‐” before the number illustrates time periods prior to recurrence. Positive numbers indicate recurrence, with treatment occurring during this time. Of note, occasionally patients did NOT receive chemotherapy immediately at recurrence as they were asymptomatic; hence, some may have initiated treatment a number of months after clinical documentation of recurrence. A, B, Illustrate cases in which sFR and CA125 display similar patterns of increase and decrease both prior to and following recurrence detection and during therapy. C, D, Demonstrate cases in which sFR levels either never return to baseline following adjuvant therapy (C) or increase before CA125 levels in the months preceding clinical recurrence. E, F, Depict cases in which CA125 levels increase while sFR levels are unchanged or decrease/lag behind CA125 changes prior to recurrence

To evaluate the use of sFR as a biomarker in surveillance, we conducted a pilot study in which we monitored sFR and CA125 in 28 patients at different time‐points spanning from post‐surgery to chemotherapy and disease recurrence. All of these patients were optimally debulked and received adjuvant chemotherapy prior to enrolling in the study; however, they were enrolled at different time‐points during their surveillance period. All 28 patients except three had platinum sensitive disease (three patients had PFS of approximately 5 months prior to recurrence). Twenty of the patients (~72%) were optimally cytoreduced at their initial surgery, and the remainder were suboptimal (28%). We observed three distinct patterns of sFR and CA125. Representative patient data are depicted in Figure 2. In the first pattern, comprising 11/28 patients (Figure 2A,B), sFR and CA125 levels mirrored each other. In the second scenario, observed in 11/28 patients (Figure 2C,D), sFR alone indicated recurrence, as its levels never returned to baseline following adjuvant therapy or increased. The increase in sFR levels was measured up to 24 months prior to recurrence in some patients. In the third scenario, illustrated by 6/28 patients (Figure 2E,F), CA125 alone indicated recurrence, with levels remaining constant (ie never returned to baseline following therapy) or increased, in contrast to normalized or even decreasing sFR levels.

## 4 DISCUSSION

Our findings suggest that sFR can distinguish between healthy controls, benign patients and those with OVCA. Furthermore, we demonstrate that sFR levels were independent of CA125 levels in the original diagnosis cohort. While a definitive threshold of sFR was not clearly identified above which OVCA could be conclusively demonstrated in the general population, as sFR increased, there was a concordant increase in OVCA risk when compared to healthy patients or benign masses. This suggests that a threshold with high predictive value could be achieved.

Our qualitative findings of differential patterns of sFR and CA125 patterns in recurrence further suggest that FR‐enriched subpopulations might be independent from CA125‐secreting populations. Since serum values likely arise from the active tumor burden, persistence of sFR at therapy completion or rising sFR during surveillance should be indicative of relatively drug‐resistant pockets of tumor cells characterized by high tumor FR expression and low CA125. Our cohort of 416 subjects constitutes one of the largest comparison groups for sFR in OVCA analyzed so far. Another important strength of our study is that, unlike other retrospective studies, we defined the background level of sFR using a large number of healthy volunteers.

The importance of serum biomarkers in the diagnosis of OVCA has grown in recent years due to an increased recognition of their utility and sensitivity.^11^ For instance, in September 2011, the FDA approved human epididymis protein 4 (HE4) as a biomarker for monitoring patients with epithelial OVCA.^12^ A “Risk of Ovarian Malignancy Algorithm” (ROMA) utilizing combined serum measurements of HE4 and CA125 demonstrated increased sensitivity in diagnosing OVCA,^13^, ^14^ particularly in the early‐stage patient group.^15^ The Tainsky laboratory has also defined specific paraneoplastic autoantibodies as potential markers for initial diagnosis and recurrent disease.^16^, ^17^

Previous research suggested that intra‐tumor heterogeneity and tumor “plasticity” might promote chemoresistance due to the presence distinct subclonal populations harboring different marker phenotypes.^18^ This could select for a subset of clones optimally suited to proliferate in particular environments, thus allowing cancer cells to overcome selective pressures such as chemotherapy.^18^ This ability of heterogeneous cancers to evolve in response to selective pressure may undermine the accuracy of single biomarkers as effective tools for cancer surveillance. For instance, although the original application of serum CA125 was for monitoring response of OVCA during chemotherapy and in detecting recurrence, this marker is plagued by poor sensitivity, thus highlighting the unmet need for additional biomarkers of recurrence.^19^ Our results further demonstrate the limitations of CA125 alone as a measure of prognostic sensitivity and suggest that additional monitoring of sFR, which can be detected up to 2 years prior to recurrence in some instances, could be beneficial. Indeed, our diagnostic cohort findings suggest that multi‐marker panels incorporating several biomarkers that take into account the different molecular and biological behaviors of cancer subtypes should greatly improve diagnostic sensitivity compared to single markers.

Our observations also agree with previously published investigations. For instance, Hori and Gambhir advanced a model simulating plasma biomarker kinetics “primed” on ovarian tumor growth and CA125 shedding data.^20^ Their conclusion postulated that a single biomarker alone was not sufficient to detect tumor growth and that a combination of biomarkers might be required to improve diagnosis.^20^ A few studies examining the expression profile of sFR in relation to OVCA aggressiveness, survival, and stratification have been published^9^, ^14^, ^21^, ^22^, ^23^ and seem to agree with this conclusion, as well. Indeed, Basel et al demonstrated the functionality of circulating FR, confirming its suitability as a biomarker of early cancer detection.^21^ O'Shannessy et al reported the largest retrospective trial to date, with 176 serous OVCA patients and measurements of sFR in conjunction with HE‐4. The AUC for sFR alone was 0.8 for “normal” versus OVCA, as compared to 0.87 in the current study.^23^ Kurosaki et al reported the only prospective trial examining sFR in newly diagnosed patients, demonstrating its potential predictive value. The reported diagnostic sensitivity, specificity, and PPV for sFR for OVCA in this trial were 59.4%, 97.9% and 97.4%, respectively, and the AUC of the ROC curve was 0.79 (marginally better than CA125). This trial was in a cohort of Japanese patients, and these results have not been validated in a large western cohort of patients. Additionally, no post‐chemotherapy and surveillance cohort was included.^9^ Finally, an ELISA‐based assay was utilized which is not as sensitive as the folate‐binding assay employed in the current study.

Collectively, our data demonstrate that sFR could improve diagnostic accuracy of OVCA. Additionally, the monitoring of both sFR and CA125 (and potentially other markers) could conceivably improve up‐front, adjuvant therapy, identifying patients who might benefit from a maintenance strategy, and also cancer surveillance, likely affecting prognosis. Our surveillance cohort is limited by the number of patients, and further studies need to be performed in order to validate this marker.

We previously reported the induction of tissue FRα by steroidal and transcription modulators,^24^ thus reducing tissue heterogeneity and increasing sFR levels which would otherwise be present at low levels. Induction of both FRα and sFR is a potentially promising approach for distinguishing between benign and malignant conditions and could provide increased sensitivity for detecting sFR, thus circumventing limitations of the current study. With this in mind, we have initiated a prospective clinical protocol to study sFR induction in both up‐front diagnosis and surveillance of OVCA to validate the use of this marker. If successful, this strategy may yield an important biomarker and therapeutic target for antibody‐drug conjugates, folate‐drug conjugates, or FR‐targeted antifolates, as well as for FR‐linked imaging modalities to monitor response to treatment and inform personalized treatment options.

## CONFLICT OF INTEREST

None declared.
