# Supplementary figures and images for: Serum folate receptor α (sFR) in ovarian cancer diagnosis and surveillance
Source: Cancer Med. 2019 Feb 13;8(3):920–7. doi: 10.1002/cam4.1944 (PMC6434204; doi:10.1002/cam4.1944)

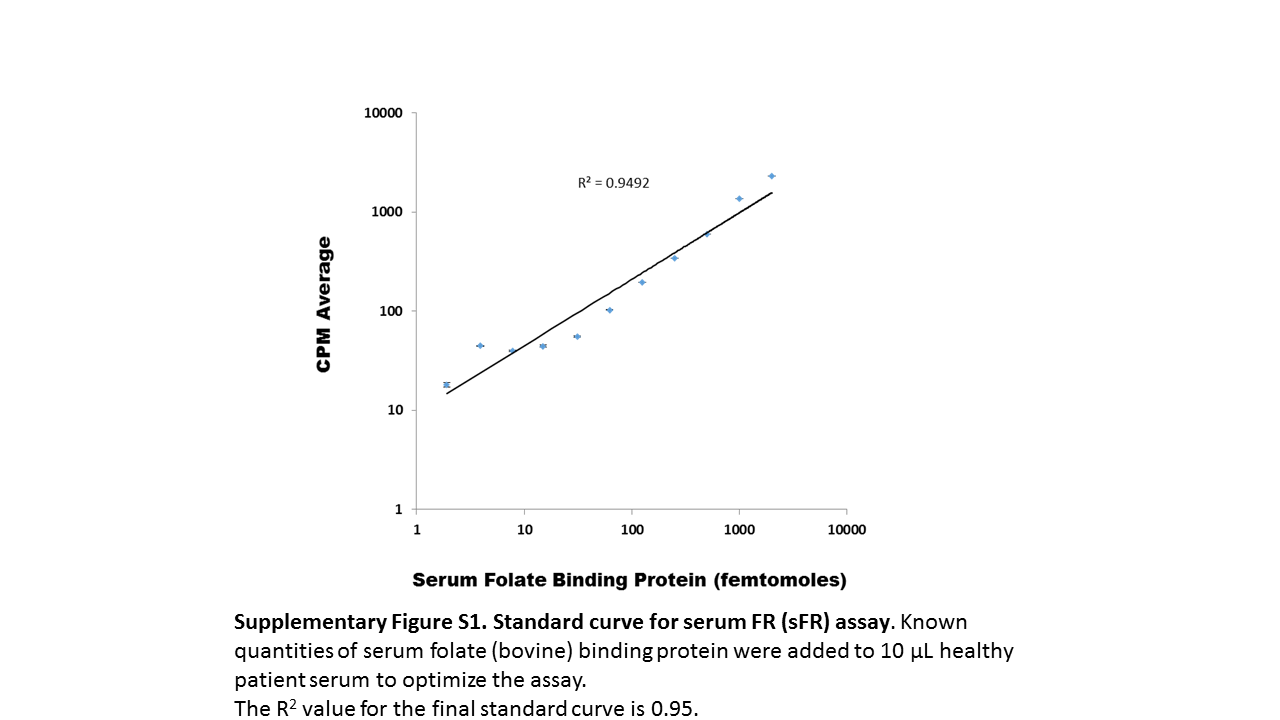

Supplement: Supplementary file 1 [file CAM4-8-920-s001.tif]

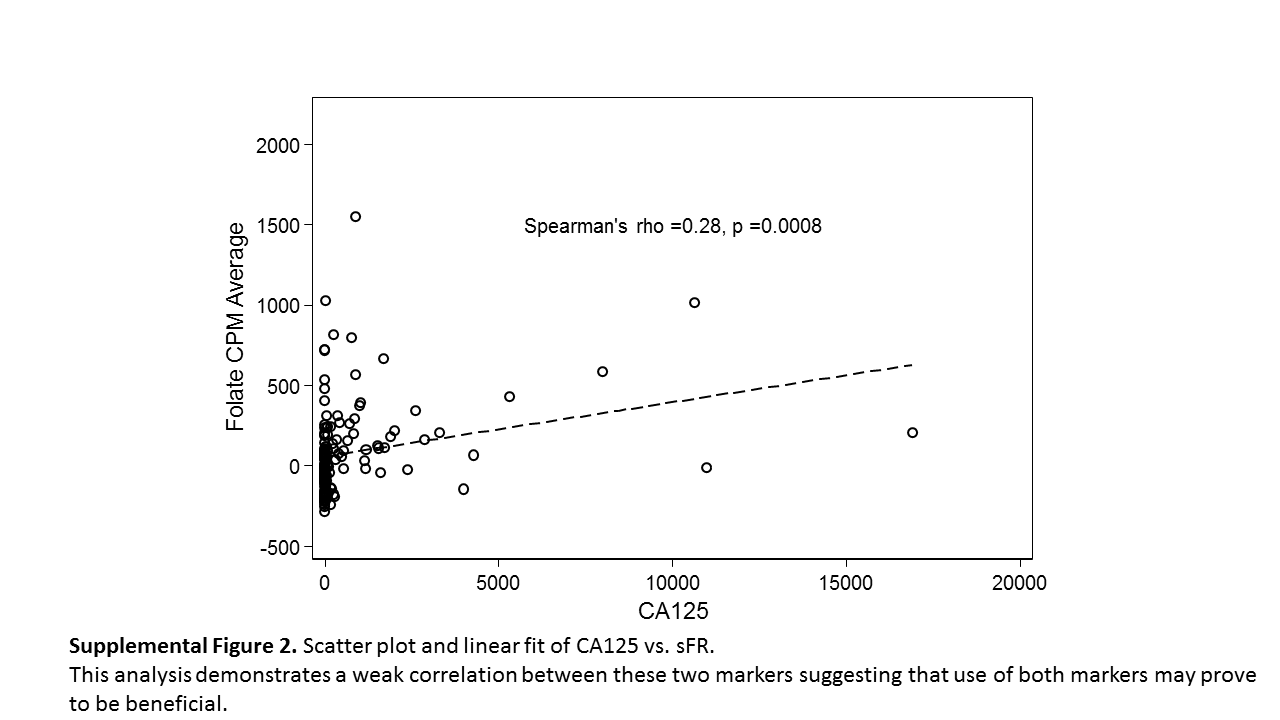

Supplement: Supplementary file 2 [file CAM4-8-920-s002.tif]
