# Supplementary material for: Serum folate receptor α (sFR) in ovarian cancer diagnosis and surveillance
Source: Cancer Med. 2019 Feb 13;8(3):920–7. doi: 10.1002/cam4.1944 (PMC6434204; doi:10.1002/cam4.1944)
Supplement: Supplementary file 3 [file CAM4-8-920-s003.docx]

**Supplementary Table S1. Cohort Demographics.** Patient clinical-pathologic characteristics for the three different patient populations for the use of sFR in diagnosis of ovarian cancer are depicted.

|  | N | Mean | 95% CI |
| --- | --- | --- | --- |
| Healthy/Benign | 172 | 66.7 | 28.2, 105.1 |
| LMP/OVCA | 194 | 236.4 | 194.4, 278.2 |
|  |  |  |  |
| Benign | 92 | 166.4 | 110.0, 222.7 |
| LMP/OVCA | 194 | 236.4 | 194.4, 278.2 |
|  |  |  |  |
| Benign | 92 | 166.4 | 110.0, 222.7 |
| OVCA | 180 | 234.1 | 191.1, 277.0 |
|  |  |  |  |
| Healthy | 80 | -48.0 | -87.3, -8.7 |
| OVCA | 180 | 234.1 | 191.1, 277.0 |

**Supplementary Table S2. sFR levels by comparison group**. CPM average for each group is depicted. These are the raw values for each group calculated with 95% confidence interval (CI). Individual histologic subtype values were also calculated and are noted within Figure 1 within the body of the paper.

|  |  | Sensitivity (95% CI) | Specificity (95% CI) |
| --- | --- | --- | --- |
| LMP/OVCA | Healthy/Benign | 71% (65%, 78%) | 65% (58%, 72%) |

1. False positive rate by group

|  | False Positive | Total |
| --- | --- | --- |
| Control (Healthy) | 6 (8%) | 80 |
| Benign Gyn | 54 (59%) | 92 |

1. False negative rate by histology

| Histology | False Negative | Total |
| --- | --- | --- |
| Serous | 37 (28%) | 132 |
| Clear cell | 5 (36%) | 14 |
| Endometrioid | 6 (29%) | 21 |
| Mucinous | 3 (23%) | 13 |
| LMP | 3 (21%) | 14 |

**B.**

| OVCA | Healthy | Sensitivity (95%CI)  91% (85%, 94%) | Specificity (95% CI)  59% (48%, 69%) |
| --- | --- | --- | --- |

1. False positive rate for controls

|  | False Positive | Total |
| --- | --- | --- |
| Control (Healthy) | 33 (41%) | 80 |

1. False negative rate for by histology

| Histology | False Negative | Total |
| --- | --- | --- |
| Serous | 11 (8%) | 132 |
| Clear cell | 2 (14%) | 14 |
| Endometrioid | 1 (5%) | 21 |
| Mucinous | 3 (23%) | 13 |

**Supplementary Table S3. False positive and negative rate of sFR by group. A: Comparing LMP/OVCA to healthy and benign.** False positive rate of healthy and benign patients by sFR for OVCA detection is noted utilizing the background cutoff determined by the healthy patients, in this example comparing healthy/benign to LMP/OVCA. The false negative rate by histologic subtype of OVCA is noted, again by sFR level. Clear cell patients had the highest false negative rate, likely due to significantly reduced numbers. **B: Comparing OVCA to healthy patients alone.** False positive rate of healthy patients by sFR for OVCA detection is noted utilizing the background cutoff determined by the healthy patients, in this example comparing healthy to all OVCA. False negative rates by histologic subtype is again noted by sFR level.
